# Cultural competence in modern global world: Applications for mental health

**DOI:** 10.1192/j.eurpsy.2021.305

**Published:** 2021-08-13

**Authors:** R. Shilko, L. Shaigerova

**Affiliations:** Faculty Of Psychology, Lomonosov Moscow State University, Moscow, Russian Federation

**Keywords:** mental health, cultural competence, global world

## Abstract

**Introduction:**

Cultural competence and related terms began to appear in the 1960s in the context of the development of civil rights movements in many countries. The importance of research of cultural competence among mental health professionals is raised with the globalization trends of the modern world, when the growth of ethno-cultural diversity, internal and external migration, temporary movement of people lead to intensification of intercultural interaction.

**Objectives:**

The current study aims to reveal contemporary tendencies in cultural competence understanding and development.

**Methods:**

Theoretical analysis and systematization of research publications in order to clarify concepts, models and applications of cultural competence.

**Results:**

The following tendencies were revealed. Cultural competence continues to attract significant attention of researchers and practitioners, especially among the mental health specialists (psychologists, psychiatrists, psychotherapists) who work with representatives of different cultures. A number of similar concepts and their components have been proposed: cultural competence, intercultural communicative competence, cross-cultural competence, cultural intelligence, cultural awareness, cultural acceptance, intercultural sensitivity, intercultural adaptation, multicultural competence, multicultural orientation. The difficulties and limitations of existing models noted: a shift of attention to a specialist, but not to a client; borrowing static and absolutistic ideas about cultures, without consideration of cultures development and interaction.

**Conclusions:**

There is a trend in contemporary global world for broad research and development of cultural competence that improve professional qualities of healthcare professionals and provide psychological assistance to representatives of different ethnic and culture groups, confessions and minorities. The reported study was funded by the Russian Foundation for Basic Research, project number 17-29-02506.

**Disclosure:**

No significant relationships.

